# Development of an App for Tracking Family Engagement With Early Intervention Services: Focus Groups and Pilot Evaluation Study

**DOI:** 10.2196/45957

**Published:** 2023-09-12

**Authors:** Liliana Wagner, Laura Corona, Nibraas Khan, Madison Hooper, Alexa Dixon, Ambar Munoz Lavanderos, Zhaobo Zheng, Nandan Sarkar, Nilanjan Sarkar, Zachary Warren

**Affiliations:** 1 Department of Pediatrics Vanderbilt University Medical Center Nashville, TN United States; 2 School of Engineering Vanderbilt University Nashville, TN United States; 3 Yale University New Haven, CT United States

**Keywords:** mobile health, early intervention, families, mobile phone, autism, focus groups

## Abstract

**Background:**

Expedient access to early intervention (EI) systems has been identified as a priority for children with developmental delays, identified disabilities, and other special health care needs. Despite the mandated availability of EI, it remains challenging for families to navigate referral processes and establish appropriate services. Such challenges disproportionately affect families from traditionally underserved communities. Mobile health apps can improve clinical outcomes, increase accessibility to health services, and promote adherence to health-related interventions. Though promising, the implementation of apps within routine care is in its infancy, with limited research examining the components of what makes an effective app or how to reach families most impacted by inequities in health care delivery.

**Objective:**

In study 1, we conducted focus groups to access a broad range of perspectives on the process of navigating the EI system, with the dual goals of identifying ways in which a patient-facing app might facilitate this process and identifying barriers to use with traditionally underrepresented and underserved groups. In study 2, focus group findings informed the development of a patient-facing app, which was subsequently tested with a pilot sample of 5 families.

**Methods:**

In study 1, the focus groups included 29 participants from 4 shareholder groups. Targeted sampling was used to recruit participants from traditionally underrepresented groups. Focus group questions sought information about barriers families experience as they navigate the EI system, ideal features of a patient-facing app designed to track family engagement with the EI system, and potential barriers. Focus group procedures were informed by the Consolidated Framework for Implementation Research framework. In study 2, a pilot app was developed. The app was tested with a sample of 5 families of young children involved in the EI system. Families provided information on app functionality and usability.

**Results:**

Qualitative analysis revealed a desire for increased communication and information about the process of accessing EI services, potential utility of an app for communication purposes, and clear recommendations for app features. Insights from focus groups were used to inform the development of the Family on Track app and related implementation supports. App features included survey customization, timing and delivery of prompts, and questions related to barriers and service satisfaction. Implementation supports include a visual guide for app installation, resources related to common family questions, and availability of study personnel to guide families through installation and provide ongoing support. Field testing provided preliminary information about app usability, including identifying future directions.

**Conclusions:**

The results of this study could support the development of a new way for the EI system to communicate and connect with families, provide families with a means to communicate satisfaction and frustration, and access the supports they need to be active participants in their child’s care.

## Introduction

### Background

Early identification and expedient access to early intervention (EI) systems have been identified as key priorities for children with developmental delays, disabilities, and other special health care needs [1,2]. Access to EI has been linked to positive long-term developmental outcomes [3-6] as well as to improvements in parental self-efficacy and family quality of life [7]. Statewide EI systems represent a key avenue through which families can access services and supports for children with disabilities or developmental delays. Part C of the Individuals with Disabilities Education Act [8] mandates that all US states maintain a system of EI services. Within these systems, any child aged <3 years with suspected developmental delays can be referred to their statewide EI system by a family member, a medical provider, or any other person in contact with the child. After referral, the child receives a developmental evaluation and may qualify to receive services to promote their development in targeted areas (eg, physical therapy and speech therapy). Unfortunately, it remains challenging for families to navigate referral processes and establish EI services [4,9]. Such challenges disproportionately impact families from rural and traditionally underserved communities [10,11].

Telehealth approaches to EI delivery have increased in recent years, hastened by the COVID-19 pandemic [12]. However, to date, limited research has examined the use of technology to facilitate family navigation of and initial access to EI services. Within other health care domains, there is a growing use of technology apps for personalizing patient care and their potential to reach a wide range of families [13,14]. It is estimated that 70% to 80% of American adults of childbearing age have access to a smartphone with connectivity and internet access, including those in low-income, rural, and racially and ethnically diverse communities [15].

Mobile health apps have the potential to reach and be well received by many individuals, improve clinical outcomes, increase accessibility to health services, and promote adherence to health-related interventions [16-19]. Mobile apps may also mitigate the barriers encountered by using traditional means of contacting families. For example, phone calls may be intrusive or inconvenient for families in comparison with prompts sent via text or apps. Questionnaires sent via mail or email are often lengthy, redundant, and usually cannot be personalized for individual families, whereas mobile apps offer the potential for brief, targeted prompts that can be personalized based on past user responses. Traditional questionnaires also rely on retrospective reporting, which may be imprecise regarding the timing of target behavior in contrast to responses given in the moment [20]. Furthermore, past reviews have documented the promise of mobile health apps specifically for traditionally underserved populations [21,22].

Although promising, the implementation of patient-facing apps within routine care is in its infancy [14,23,24], with limited research examining the components of what makes an effective app or how to reach those families most impacted by existing inequities in health care delivery. Although mobile health apps have the potential to reach and engage traditionally underserved families, it is not sufficient to simply create an intervention and expect success. Many of the currently available mobile health apps are not grounded in research and are not designed with the specific needs of their target population in mind [25].

To address these shortcomings, Baumann and Cabassa [26] proposed the use of equity-focused implementation science frameworks to successfully address health care disparities in historically underserved populations. To do so requires the involvement of shareholders from susceptible populations in the development of apps, consideration of the unique contextual factors that shape the implementation and maintenance within communities impacted by low resources, and evaluation of implementation through an equity lens.

To date, the Consolidated Framework for Implementation Research (CFIR) [27] has been used broadly in health-related implementation research [28] and increasingly in the domain of mobile health apps [23,24]. The CFIR framework comprises 5 domains: intervention characteristics, outer setting, inner setting, characteristics of individuals, and the implementation process. Within these domains are 39 constructs that support successful implementation of the intervention. CFIR is intended to be used flexibly such that researchers can identify and use constructs that are most relevant to individual interventions.

### Objectives

The purpose of this project was to access a broad range of perspectives on the process of navigating the EI system, with the ultimate goals of (1) identifying ways in which a patient-facing app might facilitate that process, (2) identifying potential barriers to its use with traditionally underrepresented and underserved groups, and (3) developing and piloting such an app with a small sample of users. This project proceeded in multiple phases, documented here as 2 studies. In study 1, the research team conducted a series of focus groups to systematically gather the perspectives of families, community providers, and health equity professionals. Focus groups sought to gather information on (1) barriers families experience as they navigate the EI system, (2) ideal features of a patient-facing app designed to track family engagement with the EI system, and (3) potential barriers affecting such an app’s use and uptake in underserved communities. In study 2, focus group themes were used to inform the development of a pilot app, Family on Track, intended to track family engagement with the EI system. We conducted a field test of the app with 5 caregivers with children currently involved in Tennessee’s statewide EI system. The intent of this field test was to demonstrate the proof of concept, specifically documenting app functionality and usability.

## Methods

### Ethical Considerations

All focus group participants were compensated for their time, and all study procedures were approved by Vanderbilt’s Institutional Review Board (#220576).

### Study 1

#### Participants

The focus group comprised 29 participants across 4 groups: 9 family members, 10 clinicians and clinic staff members, 5 community providers serving children with developmental delays and disabilities, and 5 experts in healthy equity (Table 1). The specific participant groups were selected because of their unique involvement and perspectives related to the statewide EI system (ie, families receiving services, clinicians referring to EI services, and community providers delivering EI services). Multiple participant groups were interviewed to increase the credibility of the data (ie, triangulation across data sources), an essential component of establishing trustworthiness in qualitative research [29].

**Table 1:** Participant demographics by shareholder group.

Demographics		Full sample (N=29), n (%)	Families (n=9), n (%)	Clinicians (n=6), n (%)	Clinic staff (n=4), n (%)	Community providers (n=5), n (%)	Health equity experts (n=5), n (%)
**Sex**							
	Male	0 (0)	0 (0)	0 (0)	0 (0)	0 (0)	0 (0)
	Female	29 (100)	9 (31)	6 (21)	4 (14)	5 (17)	5 (17)
**Race**							
	Asian	1 (3)	0 (0)	1 (3)	0 (0)	0 (0)	0 (0)
	Black or African American	7 (24)	4 (14)	0 (0)	1 (3)	0 (0)	2 (7)
	White	21 (72)	5 (17)	5 (17)	3 (10)	5 (17)	3 (10)
**Ethnicity**							
	Hispanic or Latinx	7 (24)	2 (7)	0 (0)	1 (3)	1 (3)	3 (10)
	Not Hispanic or Latinx	22 (76)	7 (24)	6 (21)	3 (10)	4 (14)	2 (7)

#### Families

Family members were eligible to participate if they had a child who was currently receiving services through the statewide EI system. Families were recruited through an existing clinical database and flyers distributed at university-based clinics predominately serving families from racially and ethnically diverse groups. Efforts were made to oversample families who identified as a member of a racial or ethnic minority group and families living in medically underserved areas, as defined by the Health Resources and Service Administration, using the family zip code as listed in our clinical database. This targeted recruitment was intended to capture the unique contextual factors of the traditionally underserved populations currently navigating the EI service system.

#### Clinicians and Clinic Staff

Clinician participants were professionals (ie, licensed psychologists and developmental nurse practitioners) who regularly evaluate children at risk for developmental concerns and make frequent referrals to the EI system. Half (3/6, 50%) of the clinicians were recruited from within our academic medical center and half (3/6, 50%) were recruited from external sites. The clinic staff included research assistants and family navigators working throughout our medical center, who often assist families in initiating EI services and attempt to address barriers to participation.

#### Community Providers

Community providers were eligible to participate if they worked with at least 5 children with developmental differences per week as part of community health care or educational entities. As part of their professional roles, community providers frequently referred families to the statewide EI system and provided services within the system. Participants were recruited via past involvement with professional training led by our research group and collaborative relationships with the state EI system. These professionals included developmental therapists, service coordinators, and board-certified behavior analysts.

#### Health Equity Experts

Experts in health equity research included professionals with a master’s degree or above (eg, psychologists, neurologists, developmental-behavioral pediatricians, and speech-language pathologists) who (1) were employed at an academic medical center; (2) have research and clinical interest in the areas of diversity, health equity, and health disparities; and (3) have been in practice for at least 5 years. These experts were identified through their involvement with professional organizations (eg, American Psychological Association) and partnerships with academic medical centers. These experts were included in the focus group discussions to ensure that sufficient attention was given to issues of health equity and technology use.

This project also included a partnership with a parent of a child with special health care needs and extensive experience in navigating the EI system. This parent provided guidance and perspective throughout the project, including assisting with recruitment, providing suggestions related to a potential app, and reviewing the focus group interview guides.

#### Focus Group Procedures

We conducted 7 focus groups across 4 different participant groups. All focus groups were conducted via a secure video platform, and attendance at each group varied based on participant availability (2-5 participants per group). Separate focus groups were conducted for each participant group to promote candid responses. Focus groups averaged 60 minutes in length. All the groups were audio recorded and transcribed using an institutional review board–approved transcription service. In qualitative research, there are no universal sample size guidelines for achieving results. Rather, it is recommended that data collection continue until data saturation is achieved and no new themes are being identified [30]. After conducting 7 focus groups, we found from a review of our transcripts that data saturation had been achieved.

#### Focus Group Guide

A focus group guide was developed to maintain consistency across focus groups and provide prompts to encourage robust data collection. We developed 3 iterations of the interview guides to permit tailoring of the questions to different participant groups; however, all guides followed the same format. The focus group guide was divided into 2 sections. The first part of the guide included semistructured interview questions to better understand the challenges families face in navigating referrals to the EI system and establishing services, which disproportionately impact families from underserved communities. The second half of the guide was used to elicit participant feedback on the utility of a future patient-facing app designed to track family engagement through the EI system.

To solicit feedback about an app that did not yet exist, the study team created a list of potential questions to track family engagement with the EI system that could eventually be integrated into an app. The questions were developed specifically for this purpose, in partnership with a team of EI providers, clinicians, and family navigators who currently help families access services. The questions focused on the completion of statewide EI system milestones (scheduling a developmental evaluation, creating an Individualized Family Service Plan, and initiating therapies), current receipt of child services (eg, developmental therapy, speech therapy, and occupational therapy), family satisfaction with services, and barriers experienced (Table 2 provides the sample questions). Questions were intended to capture family progress through the referral process as well as to document their satisfaction with services and any barriers encountered throughout the process. The questions were intended to be repeated serially as families move through the process of service eligibility and initiation. The questions were presented to the focus group participants in 2 different computer-based formats, each of which had the potential to be translated into a future app. Both versions included (1) the same series of questions described earlier and (2) the capacity to prompt families at preidentified intervals to answer these questions. Questions were designed to be brief and targeted (ie, families only receive questions applicable to them based on their responses to previous questions). The 2 presentation formats differed in (1) their presentation and user interface; (2) the degree of survey customization based on user responses; and (3) back-end processes for downloading, interpreting, and organizing data.

**Table 2:** Sample questions delivered via app.

Topic area	Sample questions
EI^a^ service system milestones	“Has someone from [the EI system] contacted you?” “Did your child qualify to get therapies from [the EI system]?” “Have you set a meeting with your [EI system] coordinator to set your child’s goals?”
Current services	“What therapies is your child receiving as part of [the EI system]?”
Satisfaction	“Are you satisfied with the therapies your child is receiving?” “Are you satisfied with the communication between you and [the EI system]?”
Barriers	“Do any of the following barriers apply to you and your family? Check all that apply. There are long waitlists for the services my child needs There are limited options near my home I do not have reliable transportation I do not have stable internet access for telehealth appointments or email communication Other (please describe)”

^a^EI: early intervention.

After reviewing the questions, participants were asked to share their perceptions of such an app, including its potential utility and barriers to use, both from the perspective of families (eg, digital literacy, privacy concerns, access to Wi-Fi or technology, demographic factors, time, patient education, app features, and perceived value) and clinicians, clinic staff, and community providers (eg, clinician time, perceived value, IT infrastructure, technology support, data analysis, and possibility of coordinating care with other professionals).

The CFIR framework [27] informed the development of the focus group questions. The CFIR was selected because of its ability to systematically identify and assess multilevel barriers and facilitators to guide intervention adaptations and implementation strategies. As noted earlier, CFIR contains 39 constructs and is intended to be used flexibly such that researchers select only constructs relevant to their investigation. Constructs from 4 domains within the CFIR framework were selected based on their relevance to our population of intended users and the specific features of our product (ie, app). The four domains included (1) intervention characteristics (eg, relative advantage of the app over existing tools, design and adaptability of the app, and complexity of use), (2) outer setting (eg, consideration of patient needs and available resources), (3) inner setting (eg, compatibility with existing processes and workflow and shareholder values, motivation for change among shareholders, available resources to facilitate implementation, and ease of access to training and information on the use of the app), and (4) characteristics of individuals (eg, individuals’ attitudes toward the app and their belief in their ability to use the app successfully). In addition to the CFIR-related questions (Table 3), we asked specific questions related to the features of a future app (eg, How frequently would you like to receive reminders to complete questions about your engagement with the EI system? How much would it bother you to be asked the same question at multiple time points? Is this language consistent with the language you use to describe EI services?).

**Table 3:** Included Consolidated Framework for Implementation Research (CFIR) constructs and related interview questions.

CFIR construct		Interview question
**Intervention characteristics**		
	Intervention source	“How important is it that you are familiar with the app?” (Probe for name recognition of MyCap vs Vanderbilt University Medical Center-developed Family on Track)
	Relative advantage	“What if any benefits could use of this app have over your current systems for tracking family engagement in EI^a^ services?”
	Adaptability	“What changes would you need to make so this app works for your family/your patients/your clients?”
	Complexity	“How complicated is the language used throughout the app? Is it clear what would be expected of you and your patients when completing this app?”
	Design quality	“What design qualities are most important in an app like this? What features of the app do you like and dislike?”
**Outer setting**		
	Patient needs and resources	“Would an app like this meet the needs of your patients? What direct benefits would families see from use of this app? What would make a family most likely to use this app?”
**Inner setting**		
	Compatibility	“How would an app like this fit into your clinic processes or workflow?”
	Tension for change	“How satisfied are you with your current ways of tracking family engagement? Do you feel that you are successfully able to navigate the EI system at this time?”
	Available resources	“What resources would you anticipate needing to encourage uptake?”
	Access to knowledge and information	“What kind of training would you need to feel comfortable using this app and instructing families to use this app?”
**Characteristics of individuals**		
	Knowledge and beliefs about the intervention	“Do you think this app will be an effective way to track family engagement with the EI system?” “Do you believe this app could be easily implemented within the EI system?”
	Self-efficacy	“How confident do you feel about your ability to use an app like this? How confident do you feel about assisting families with use of this app?”

^a^EI: early intervention.

#### Moderators

The first or second authors served as moderators for each focus group. To maintain consistency across the focus groups, the moderators reviewed the focus group guide together and discussed phrasing and prompts for specific interview questions. One component of establishing trustworthiness in qualitative research is attempting to ensure confirmable findings [31]. In essence, the data collected should reflect the true opinions of the study participants and should not be influenced by the biases or assumptions of the data collectors. In advance of the focus groups, both moderators also reviewed and discussed guidelines for focus group moderation, which included withholding personal opinions, attempting not to interrupt participant thought processes, ensuring that all participants were given the opportunity to share their thoughts, summarizing participant responses to ensure the accuracy of interpretation, and maintaining a neutral affect and impartial attitude to encourage open responses. Importantly, both moderators have graduate training in clinical interviewing and regularly provide therapeutic services to families and children; thus, they are aware of the clinical skills and behaviors needed to cultivate a warm, nonjudgmental environment. The fourth author attended 25% (1/4) of the focus groups to record the sessions and take notes. As the fourth author was also responsible for coding the transcripts, her notes were used to provide context when coding and analyzing the qualitative data.

#### Data Collection

At the beginning of each focus group, the focus group moderator informed participants that the focus groups would be recorded for transcription purposes and that all attempts would be made to ensure confidentiality of the data. Participants were encouraged not to share their full names or the names of their children if applicable. Once verbal consent was obtained, the moderator reviewed the guidelines for the focus groups, including not interrupting others, respecting others’ views and experiences, and not sharing focus group information with outside individuals. The moderators followed the interview guide during the focus groups. Questions and follow-up prompts were asked in a flexible manner to follow the flow of the conversation. The conversation surrounding each question continued if new information was being added and until each participant had the opportunity to share their opinion.

#### Coding Procedures

Following the focus group discussions, transcripts were coded to reveal themes and subthemes that emerged across participant groups and could be reliably identified by multiple raters. The coding of focus group transcripts was completed using a content analysis and predominately deductive approach guided by the CFIR. Specifically, a codebook was developed a priori by the first author based on the CFIR domains and constructs. Within each domain, the first and third authors developed a set of code concepts with accompanying definitions based on anticipated themes after reviewing the transcripts. We were also open to the possibility that new themes could inductively emerge from the data. After coding the initial transcript, the first author met with the third and fourth authors to remove duplicate codes and to create a master codebook. The third author coded all transcripts in Microsoft Excel, with each row of data representing a separate quotation that could be assigned up to 5 codes. To ensure rigor in coding, 25% (1/4) of the transcripts were double coded by the fourth author. The areas of disagreement were reviewed and discussed until 100% consensus was achieved. When necessary, the first author was involved in discussions to help clarify responses and assist in resolving coding differences. The coded interviews were imported from Excel (Microsoft Corporation) into SPSS (IBM) for sorting analysis. Direct quotations were provided to connect the results to the raw data.

### Study 2

#### App Development

Qualitative focus group data analysis was used to inform the development of an app in partnership with the university department of engineering. This pilot version of the Family on Track app focused on tracking family engagement with the EI system by prompting families at prespecified time points to complete brief questions about the EI referral process and any barriers encountered. Table 4 maps themes identified by the focus groups to the related features of the app. Given the preliminary nature of this work, not all focus group feedback could be incorporated into the app itself. Some focus group feedback was addressed through related implementation supports, such as written or web-based information shared with families at study onset (Table 4).

**Table 4:** Mapping focus group feedback to app design and implementation.

Domain and nested construct			Focus group theme		Strategy for addressing focus group feedback
**Outer setting**					
	Patient needs and resources	Seeking information about EI^a^ service system		Implementation support materials: visual timeline for EI services, contact information for EI system, information on types of therapies offered, and information on child development and goal setting	
**Inner setting**					
	Tension for change	Provider desire for more information about family barriers and needs		Within app: families answer questions about barriers they are experiencing	
	Available resources	Smartphone capability		Not addressed in this study	
	Access to knowledge and information	Need for accessible technology support		Implementation supports: technical support available to download the app and throughout the study period	
**Characteristics of individuals**					
	Knowledge and beliefs about the intervention	Power for families		Not addressed in this study	
**Intervention characteristics**					
	Relative advantage	Frequent and predictable information		Within app: sending prompts at regular intervals to allow families to comment on their progress and satisfaction with services	
	Adaptability	Responsiveness to family needs		Not addressed in this study	
	Design quality	Customization and simplicity		Within app: simple language developed with shareholders and customized questions based on individual user responses	

^a^EI: early intervention.

A cross-platform app (ie, Family on Track) using Flutter was developed with a Firebase back end, a Google-developed, NoSQL-based real-time cloud database. Flutter, developed by Google, is an open-source software development kit that is used to develop cross-platform apps with 1 codebase. With this tool, 1 code base can be used to develop for Android, iOS, Linux, macOS, etc. Family on Track can be installed and used on both Android and iOS devices.

**Figure 1:**
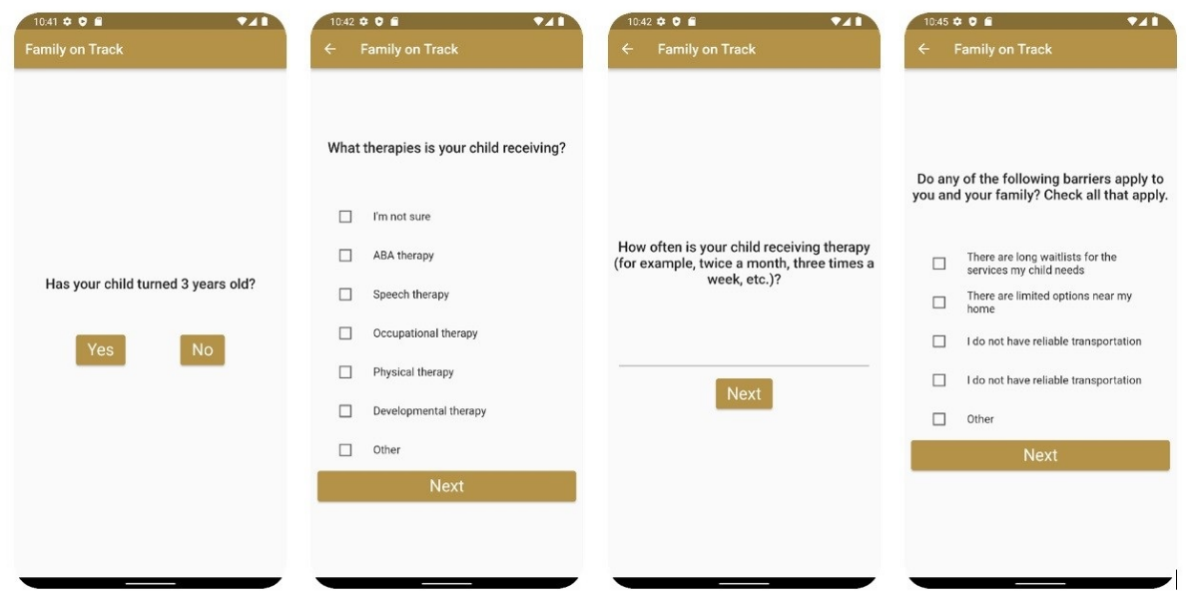
Screenshots of the Family on Track app. ABA: applied behavior analysis.

The app allows secure data collection through a customized state machine that identifies relevant questions based on prior app interactions (ie, caregiver responses) and has the capacity to recall responses given by users at prior time points to ensure that families are not asked repeated questions. The state machine was built to be modular and to adjust the flow of logic in real time based on the answers provided by the users. Individualized real-time customization ensures that the questions are personalized, leading to a short completion time. As described earlier (Table 2), the questions were initially developed in partnership with a team of EI providers, clinicians, and family navigators who currently help families access services and then revised based on insights gathered through focus groups. The questions focused on (1) communication with the EI system, (2) child involvement in therapies, (3) barriers to service access, and (4) family perceptions of their current services. Figure 1 shows screenshots of the app.

To prompt users to answer questions, they receive push notifications on their phone through an automated, fixed time schedule (1) if they have not completed their questions and (2) at the next prespecified time point. Users receive 2 reminder notifications (ie, 24 hours and 48 hours after the initial prompt to answer questions) if they have not completed the questions within this time frame. Once the user has answered their questions, the automated system will send out another push notification alerting when it is time to provide another update about their progress (ie, answer a new set of questions). Both the reminders to complete and the start of the next set of questions are determined without human intervention through an automated cloud function in Firebase, which runs every day. With this automation, we developed a fully independent surveying system that will only move forward once the user has completed all prerequisite steps.

#### Field Testing

We conducted a field test of the preliminary Family on Track app with a sample of caregivers (n=5) with children currently enrolled in their statewide EI system who participated in a developmental evaluation through a large academic medical center after being referred because of concerns regarding development. Caregivers were eligible to participate if they (1) had a child aged between 12 and 36 months who participated in a comprehensive developmental evaluation, (2) received a recommendation to initiate services through the EI system, (3) had a primary participating caregiver with access to technology (eg, phone or tablet with internet connection and ability to download apps), and (4) had a primary caregiver with sufficient facility with English to participate in the procedures and complete study measures. Children were aged between 24 and 36 months at enrollment (mean 31, SD 4.409 months). All the children were male, and all the caregivers were female. Table 5 provides additional demographic data.

**Table 5:** Study 2 participant demographics and app use (n=5).

			Full sample		Participant number								
					1		2		3		4		5
**Caregiver sex, n (%)**													
	Male	0 (0)		N/A^a^		N/A		N/A		N/A		N/A	
	Female	5 (100)		N/A		N/A		N/A		N/A		N/A	
**Child sex, n (%)**													
	Male	5 (100)		N/A		N/A		N/A		N/A		N/A	
	Female	0 (0)		N/A		N/A		N/A		N/A		N/A	
Child age at enrollment (months), mean (SD)			31 (4.409)		24 (—^b^)		27 (—)		33 (—)		33 (—)		36 (—)
**Race, n (%)**													
	Black or African American	1 (20)		0 (0)		0 (0)		0 (0)		1 (20)		0 (0)	
	White	4 (80)		1 (20)		1 (20)		1 (20)		0 (0)		1 (20)	
**Ethnicity, n (%)**													
	Hispanic or Latinx	2 (40)		0 (0)		1 (20)		0 (0)		1 (20)		0 (0)	
	Non-Hispanic or non-Latinx	3 (60)		1 (20)		0 (0)		1 (20)		0 (0)		1 (20)	
Completed prompts, mean (SD)			4.4 (1.356)		N/A		N/A		N/A		N/A		N/A
Completed prompts (n=6), n (%)			N/A		5 (83)		6 (100)		4 (67)		5 (83)		2 (33)
Reminders to complete prompts, mean (SD)			2.6 (1.497)		N/A		N/A		N/A		N/A		N/A
Reminders to complete prompts, n			N/A		3		0		4		2		4
Average time to complete prompts (seconds), mean (SD)			51 (14.221)		34 (18.416)		62 (40.648)		39 (21.545)		67 (62.765)		N/A
Fidelity (n=6), n (%)			N/A (83)		6 (100)		5 (83)		6 (100)		3 (50)		N/A

^a^N/A: not applicable.

^b^Not available.

Each family completed informed consent procedures with a member of the study team via a Health Insurance Portability and Accountability Act–compliant teleconferencing platform on the day of enrollment. Once enrolled, families were given instructions for downloading the app onto their devices and were emailed (1) a demographic questionnaire via REDCap (Research Electronic Data Capture; Vanderbilt University) and (2) a list of resources and supports related to child development and the statewide EI system that were generated during focus group discussions. Once enrolled, families responded to app-delivered prompts (ie, customized questions) related to service access and use at 6 time points over the course of 4 months (ie, at study initiation and 15, 30, 60, 90, and 120 days after study initiation). Families were notified to complete the questions via push notifications delivered by the app. To test the usability, a study coordinator reached out after the first prompt was scheduled to be sent (1) to prompt completion and (2) to determine if the participant received the prompt as scheduled. If the participants did not complete the prompt, the study coordinator sent another email approximately 24 hours later (ie, 48 hours after the first prompt was scheduled).

Each family was called by a member of the research team at one of the prespecified time points that were selected randomly and differed across families. During the calls, families were first asked to open the app and answer their next set of questions as they would on their own while talking aloud about their experience. A member of the study team interviewed caregivers using a semistructured interview guide to better understand the usability and accessibility of the app. Families were verbally asked questions they had previously answered within the app to obtain an estimate of the fidelity with which they were using the app. At the conclusion of the 4-month period, families were emailed a questionnaire through REDCap to assess caregiver perceptions and satisfaction with the app, including ease of use, clarity of instructions, timing, perceived value, and satisfaction with the services received. Caregivers were also asked questions related to possible barriers to use (technology issues and privacy concerns) and appropriateness for their specific needs. The questionnaire provided opportunities for providing open-ended feedback.

## Results

### Study 1

#### Overview

The focus group results, including barriers and facilitators, were organized according to the CFIR constructs (Table 6). Participant quotes were provided to support theme selection.

**Table 6:** Focus group themes and exemplary quotes.

Domain and nested construct			Theme		Exemplary quotes
**Outer setting**					
	Patient needs and resources	Seeking information about EI^a^ service system		“I’ve had a couple of families recently who seemed kind of confused even once they get the referral about what they’re being referred to or why.” “Like, knowing what my responsibility was and what was the responsibility of [the Part C system]. Having that differentiation is very helpful because... [I didn’t know] if I was supposed to be working toward something...”	
**Inner setting**					
	Tension for change	Provider desire for more information about family barriers and needs		“I think just confirming that they’re in therapy and the types of therapy that they’re getting helps us check off that box that, okay, we are getting the intervention that we need versus like, ‘Oh my God, it’s been three or four months. We still aren’t in any therapies, and we’re still developmentally delayed. We need all hands on deck to help this family.’”	
	Available resources	Smartphone capability		“We have to make sure that people have enough data storage, and we have to make sure that they have the types of phones that can do these functions and also the skill.”	
	Access to knowledge and information	Need for accessible technology support		“Just based on my experience with... signing families up [for services] and creating an online account... it was much more complex and complicated and took like an hour every time. But I usually found that when I would do it with families, it was much more helpful if I was sitting there with them and could walk them through it...”	
**Characteristics of individuals**					
	Knowledge and beliefs about the intervention	Power for families		“It would be great to have that place that we could go to put those questions down when we’re thinking about something. That almost, not like a journal or a diary, but I’m thinking patient portal type thing that’s individualized for us.” “I would love it if there is a way—because we collect lots of data about the child’s progress, if there was a way that the family could visualize that... just a way to help them keep track of where they’ve been, what they’re accomplishing, and moving forward...not waiting for someone else to give them that.”	
**Intervention characteristics**					
	Relative advantage	Frequent and predictable information		“You say, ‘You had reached out. We noticed you don’t have an IFSP. Look at these things. Are you still on track? Do you want to pursue that referral again?’ And just kind of send notifications back through the app to the family just to touch base on where they are developmentally.”	
	Adaptability	Responsiveness to family needs		“I mean, the goal is really to make sure families get the help that they need. ...So, if the app can help ensure my child can get the services that they need... I see most families trying it because it really is challenging for most families to get what they need for their child.”	
	Design quality	Customization and simplicity		“[It would be nice to] kind of minimize or tailor the questions each time versus it being the same set of questions over and over again because they may get some question fatigue from answering the same questions over and over again.” “One thing that stands out to me right off is just the terminology ‘Part C.’ I don’t think families really grasp that aspect of it. I think that terminology may confuse some of the families. When you get more technical, I just feel like that just kind of goes in one ear and out the other. And so I think it just adds a level of confusion to the whole process.”	

^a^EI: early intervention.

#### Outer Setting

Respondents noted that families experienced a general lack of information regarding the state EI system and the services it provided. Specifically, several respondents reported both confusion around navigating the system and a lack of understanding about why their children were referred for specific services and the purpose of those services. This is compounded by the sense of overwhelm many parents experience after learning about their child’s delays or developmental diagnosis:

The process of navigating the whole system, it’s just confusing in general. It’s confusing for anybody.

P010

So, when we got the diagnosis, it was naturally just overwhelming... And your instinct, I think, as a parent, is, “Okay, what do we do now?” And we, frankly, had no idea.

P003

Contributing to the confusion is long wait times with limited communication, during which parents wondered if there was more they should be doing. Many families reported a desire for interim communication, in which state EI system providers could suggest things that families could begin to address on their own while waiting for services to begin. In addition, parents expressed a desire for a visual timeline to track their progress through the system and better understand everyone’s roles and responsibilities:

Big delays from getting the referral, so the referral from the pediatrician goes right in; they get a call, they get evaluated. I can see the report or the evaluation, they were found eligible, but then no services were started. So, the slowness of getting the therapies that we recommend, even if all participants feel like it’s warranted and eligible for it, is a challenge.

P021

I think that would be very beneficial if you gave links to, like, what we could be doing in the meantime while we’re waiting for things... Instead of that time that you’re waiting is just kind of like wasted time.

P002

Parents expressed frustration with having to constantly reach out to service coordinators and worried that their repeated attempts at communication bothered the EI system staff members. Parents indicated that they would appreciate a way to easily communicate with their provider in between visits, as opposed to searching for an appropriate person to contact:

It was always having me to try to reach out and find information from a person... I felt like I was bothering them... And it was, that was the frustrating part, of me having to reach out.

P001

Families reported continued frustration after being contacted to begin services, as they felt a responsibility for helping to select their child’s intervention goals without having the requisite knowledge of child development:

I just got goals given to us. Like, they brought it already filled out and they were like, “These are going to be his goals.” And it just kind of... threw me off. Like, I couldn’t actually choose what we were going to be working on. So, that would’ve been very helpful, like a template of this is what it could be. And it would’ve made me want to speak up about, “Hey, I don’t think this goal suits my son. What about something like this?”

P001

It was a lot of information all at once in a world that we had no familiarity with at all. Which, I think, a lot of us are in the same place.

P003

Finally, respondents reported that it would be beneficial to have an easy way to share their satisfaction with the services they are receiving and their frustrations or barriers they are experiencing as they navigate the system:

How they feel about the services, too, if I feel like this service is not going really well, or sometimes families are afraid to say that to a service coordinator or afraid to say that to a specific therapist... But maybe the app can just say, “Hey, how do you feel like this therapy is going?”...Then that’s information for the service coordinator, too, before they even walk in like, “Hey, talk to me about OT,” or, “Do you want to just drop this service? Do you want to find another provider? What can we do to help build that relationship or restore that relationship with that provider?”

P020

#### Inner Setting

##### Tension for Change: Provider Desire for More Information About Family Barriers and Needs

Just as families expressed a desire for increased information and communication from the service system, both referring clinicians and EI providers expressed dissatisfaction with the level and type of information they receive from parents as they progress through the service system.

Clinicians and EI providers also reported that they would like to be able to identify specific barriers families are experiencing, both in initiating services and in progressing through the system. Providers also expressed that they would appreciate feedback regarding the quality of their services, so they could use that information to tailor their communication and treatment approach with individual families. For example, 1 respondent stated the following:

Just to increase the quality of my services knowing, “Okay, this family might need more support than what I am giving them,” or another family, “She shares a lot of stuff. I feel like the services are going really well.” Then that’s great. We’ll continue on that track for that family. But to kind of increase the quality of our services by knowing—having that data.

P020

##### Compatibility: Existing Familiarity With Smartphone-Based Communication and Information

When asked how an app to track family engagement with services would fit into normal clinical processes, respondents reported that families are already familiar and comfortable with smartphone-based communication. For example, families often text with EI providers to schedule appointments. Other parents acknowledged that they are currently using mobile health apps to navigate their child’s medical records, make appointments, and message their providers.

##### Available Resources: Smartphone Capability

Although smartphone and mobile app use was largely ubiquitous across all shareholder groups, respondents shared that although many people have a phone, it is important to recognize that some have limited data storage capabilities and limited access to the internet:

A large portion of people usually do have a phone, but there are a lot of people who... don’t have the data or the Internet.

P010

##### Access to Knowledge and Information: Need for Accessible Tech Support

Several respondents recognized that there may be unavoidable and unpredictable technological difficulties that will arise, and having simplified, easy-to-access tech support and instructions would ensure that all families are able to access the benefits such an app would provide:

Also, installing it is a big issue. Because sometimes a lot of them, they just don’t have enough data on their phone. So, it would be important to have an IT person or a number they could call at the beginning if they’re having trouble. Or a QR code for installation would be super helpful.

P027

#### Characteristics of Individuals

All shareholder groups expressed that the use of an app would empower families by providing them with a better understanding of the EI system as well as a consistent place to access and track their child’s information. One EI provider commented the following:

And I would love it if there is a way—because we collect lots of data about the child’s progress, if there was a way that the family could visualize that... just a way to visually help them keep track of where they’ve been, what they’re accomplishing, and moving forward, when they—at their fingertips, not waiting for someone else to give them that.

P016

#### Intervention Characteristics

##### Relative Advantage: Frequent and Predictable Communication

Respondent feedback indicated that several families experience irregular communication with EI providers. One potential advantage of this app would be the facilitation of frequent and predictable communication with clinicians and EI providers. For example, the app could contact families at specified intervals to collect information regarding their progress within the EI system.

##### Adaptability: Responsiveness to Family Needs

It became clear that to incentivize families to use the app, it will have to offer a solution to barriers frequently faced within the EI system in addition to simply tracking a family’s progress. Respondents suggested several features that would enable the app to be more responsive to family needs. For example, respondents indicated that families would benefit from explicit definitions and descriptions of the different therapies to which their children are referred:

I wonder also if there’s anywhere that you can put, like if you can click on the word or have another place in the app that kind of explained what early intervention services are in a simplified way, kind of like a—a glossary.

P014

##### Design Quality: Customization and Simplicity

Respondents across all shareholder groups collectively emphasized the importance of customization regarding surveys and questions that families will be prompted to answer throughout the app:

I definitely think that if... they have to keep on answering the same question, I think families would probably get frustrated. I think the customization would make a big difference in compliance.

P014

On the basis of the respondent feedback, prompts to complete questions about their engagement with the EI system should ideally be sent out at 2- to 4-week intervals. It is important that the prompts are not too close together, as this has the potential to make families feel bad that things are not progressing at a faster rate and subsequently make them less motivated to follow through with intervention services:

We know that things take weeks between, so the feeling of disappointment of having to say, “No,” over and over and over, “I still don’t have this together,” would be make me feel bad.

P021

Respondents also emphasized the importance of avoiding technical language and acronyms*.* Instead, the respondents recommended that the app use descriptive, lay language and built-in definitions for those who want them:

Like a question mark, you know, when you’re filling out things and then if you don’t know what the term means, you can press it and they can have a quick blurb [or description]... something in layman’s terms that can kind of explain it just to make sure they don’t say no when they really have [it] or something like that.

P014

Finally, respondents also reported that the app should be visually appealing and friendly:

I think really investing in it being visually appealing... that it’s very warm and inviting visually.

P016

### Study 2

On average, participants completed 73% (4.4/6; range 33.3%-100%) of the prompts across the course of the study. It took families an average of 51 (range 10-127) seconds to complete each set of questions. Overall, 80% (4/5) of the participants required at least 1 email reminder to respond to their prompts, with an average of 2.6 (range 0-4) reminders across participants. The average agreement between caregiver responses recorded on the app and those provided during the interview with a study team member (ie, fidelity) was 83.3% (range 50%-100%).

All participants thought the Family on Track app was easy to use, the questions were understandable, the amount of time to answer the questions was acceptable, and the timing of the reminder prompts was acceptable. Overall, 40% (2/5) of participants identified the technical features of the app that they disliked (eg, difficulty logging in and failure to update the new set of questions). One family (participant 3) required initial support logging in and then required technical support to force prompt delivery at 1 time point, as they did not receive a new prompt at the expected time. Another family (participant 4) also needed technical support to force prompt delivery at 2 time points. Moreover, 1 family (participant 5) had ongoing technical problems accessing and completing the surveys that required continuous communication with the study coordinator and a web-based meeting with the app’s engineer. Owing to these issues, the participant completed only 2 prompts. Despite technical difficulties, all families thought the app was helpful, even in its pilot version.

## Discussion

### Principal Findings

This study used CFIR-informed focus group methodology and field testing to develop and pilot a patient-facing app to track family engagement with EI services. The analysis of qualitative data from focus groups highlighted several themes, including (1) a collective desire for increased communication with the EI system, information about accessing EI services, and a way to track their progress through EI service system milestones; (2) the ubiquity and potential utility of a mobile app for these purposes; and (3) recommendations for features of such an app. These themes were used to inform the development of the Family on Track app and related implementation supports for app use that were field tested with 5 caregivers of children currently receiving services through the statewide EI system. The participant feedback also indicated several potential future directions for further studies.

### Potential Benefits and Utility of an App

Across all focus groups, participants believed that a mobile health app capable of tracking family engagement with EI services would benefit families and providers alike, including addressing challenges within the current system. Families reported feeling confused and overwhelmed by the EI system, voicing uncertainty over the selection of appropriate services and child treatment goals, limited information about service system timelines, and long waiting periods. In turn, providers reported frustration with the lack of information about a family’s progression through the EI system. They voiced the need for specific feedback about barriers families experience as well as family satisfaction (or dissatisfaction) with the system to better tailor their services.

The focus group participants described that an ideal app would have several features, including the capacity to track progress and involvement in the EI system using customized prompts and questions, the ability to answer frequent family questions about the EI system and child development, and the capacity for 2-way communication with EI providers and staff. Participants indicated that questions delivered through the app must feel directly relevant, brief, and not repetitive. Specifically, families wanted the app to be capable of storing prior responses and adjusting subsequent questions based on that information. In addition, families reported that it would be important for an app to have some flexibility in the timing of their prompts (eg, not issuing a prompt at a consistent, potentially inconvenient time every day). All groups stressed that the language used throughout the app would have to be simple and descriptive, avoiding acronyms or unfamiliar terms. Above all, participants reported that the app would have to provide clear utility for both families and providers. That is, families would be more likely to use an app that provided information and resources, as opposed to providing data only to EI providers.

### App Creation

Family on Track, the app resulting from this process, incorporated several of these shareholder insights and suggestions. In this pilot app, families answer targeted questions focused on accessing EI services, with questions tailored at each time point based on their previous responses. Questions focus on service engagement and provide opportunities to endorse or describe barriers encountered (eg, reliable transportation, waitlists, and stable internet access) and overall service satisfaction. The pilot version of this app was not able to accommodate 2-way communication, and many focus group participants desired to ask and answer questions. Therefore, implementation supports were designed for use together with Family on Track to address families’ desire for information and resources. Supports include a visual guide for app installation, resources related to common family questions regarding child development and the EI system, and availability of study personnel to guide families through installation and answer questions about the app in an ongoing manner.

### Field Testing

To gauge the usability of the app, we field tested Family on Track with 5 caregivers to collect initial data on participant retention, adherence, and fidelity related to the use of the app. Most participants (4/5, 80%) completed 4 out of the 6 prompts across the 4-month period and reported that the app was easy to use and understand. Fidelity was adequate, suggesting that the participants understood the language and content of the questions. Field testing revealed some technical issues within the app. Although these issues can be addressed quickly by the study team and engineering support, it is likely that technical support will be an essential component of any future version of this app. When asked how the app could be more helpful to families, participants’ responses were consistent with the focus groups (eg, bidirectional communication between family and EI providers, immediate delivery of resources mapped to identified barriers, and inclusion of more visual supports throughout the app). Despite the absence of these individualized, interactive features, the participants still reported that the app was helpful for families.

### Limitations

This study was exploratory, with the intention of uncovering family experiences and identifying contextual barriers and facilitators to using an app to track family engagement with the EI system. This subsequently informed the creation of a pilot app that was field tested with a small number of caregivers. Although efforts were made to recruit a representative sample of participants, the data that informed the development and field testing of this app reflected the opinions and experiences of a relatively small number of individuals. Furthermore, as is the case with many focus groups, some individuals spoke more than others, despite efforts to encourage group participation. Furthermore, the characteristics of the moderators, both White women in their 30s with advanced degrees, may have impacted data collection in ways that were not measured in this study.

The scope of this study limited the degree to which some participant suggestions could be incorporated into the preliminary version of the app. As noted earlier, an ideal app would allow for 2-way communication between families and providers, which was not accomplished with this version of the app. Some participants also envisioned features such as an interactive timeline that could be accessed within the app, allowing families to track their child’s progress through the service system milestones and plan for future events. Families also expressed a desire for information about appropriate developmental milestones, so that they could be more active contributors when it was time to set goals and track their child’s progress.

To address some of these limitations, efforts were made to create supporting materials to supplement the app and help families access some of the information they desire. Specifically, resources outside the app were created to visually depict EI service system milestones and expected timelines, to direct parents to evidence-based information about child development and developmental milestones, and to connect parents with existing EI resources related to therapies and intervention services. Despite receiving these materials at study onset, the caregivers who participated in field testing still reported that individualized resources and recommendations delivered *within* the app would be most desirable.

In this study, the use of mobile apps and smartphones was ubiquitous across shareholder groups, suggesting that Family on Track could be easily integrated within families’ lives. However, respondents cautioned that despite near-universal access to the technology, some families may not have enough storage on their devices for the data that such an app would require. Furthermore, no single technology is likely to reach all families, and it is possible that families from the most disadvantaged groups may be unable to access this type of app. Continuing to tailor strategies for reaching individuals from diverse backgrounds and with diverse needs should be the focus of ongoing research.

### Future Directions

In focus groups, several parents essentially described a full-service, interactive platform in which parents can communicate back and forth with EI providers and provide real-time feedback on a child’s progress toward their individual goals. Although the current version of the app does not facilitate 2-way communication with providers, we acknowledge this as a crucial aspect that will influence future planning. Future work could deploy an updated version of the Family on Track app with a larger group of families and collect data on participant retention, adherence, and fidelity related to the use of the app. It would also be helpful to examine family-related factors that might impact acceptance (demographics, digital literacy, perceived usefulness, and perceived ease of use) and measure key implementation outcomes (acceptability, appropriateness, feasibility, and sustainability) at the patient and system levels. Ultimately, the results of this study could support the development of a new way for the EI system to communicate and connect with families, providing families with a means through which to communicate their satisfaction and frustration, and, through the supporting materials, access the supports they need to be more active participants in their child’s care.

## Abbreviations

CFIR: Consolidated Framework for Implementation Research
EI: early intervention
REDCap: Research Electronic Data Capture
